# Artificial Intelligence-based methods in head and neck cancer diagnosis: an overview

**DOI:** 10.1038/s41416-021-01386-x

**Published:** 2021-04-19

**Authors:** Hanya Mahmood, Muhammad Shaban, Nasir Rajpoot, Syed A. Khurram

**Affiliations:** 1 Academic Unit of Oral & Maxillofacial Surgery, School of Clinical Dentistry, University of Sheffield, Sheffield, UK; 2 Department of Computer Science, University of Warwick, Coventry, UK; 3 Unit of Oral & Maxillofacial Pathology, School of Clinical Dentistry, University of Sheffield, Sheffield, UK

**Keywords:** Head and neck cancer, Head and neck cancer

## Abstract

**Background:**

This paper reviews recent literature employing Artificial Intelligence/Machine Learning (AI/ML) methods for diagnostic evaluation of head and neck cancers (HNC) using automated image analysis.

**Methods:**

Electronic database searches using MEDLINE via OVID, EMBASE and Google Scholar were conducted to retrieve articles using AI/ML for diagnostic evaluation of HNC (2009–2020). No restrictions were placed on the AI/ML method or imaging modality used.

**Results:**

In total, 32 articles were identified. HNC sites included oral cavity (*n* = 16), nasopharynx (*n* = 3), oropharynx (*n* = 3), larynx (*n* = 2), salivary glands (*n* = 2), sinonasal (*n* = 1) and in five studies multiple sites were studied. Imaging modalities included histological (*n* = 9), radiological (*n* = 8), hyperspectral (*n* = 6), endoscopic/clinical (*n* = 5), infrared thermal (*n* = 1) and optical (*n* = 1). Clinicopathologic/genomic data were used in two studies. Traditional ML methods were employed in 22 studies (69%), deep learning (DL) in eight studies (25%) and a combination of these methods in two studies (6%).

**Conclusions:**

There is an increasing volume of studies exploring the role of AI/ML to aid HNC detection using a range of imaging modalities. These methods can achieve high degrees of accuracy that can exceed the abilities of human judgement in making data predictions. Large-scale multi-centric prospective studies are required to aid deployment into clinical practice.

## Background

### Head and neck cancers: incidence and diagnosis

Head and neck cancers (HNC) comprise a heterogeneous group of cancers, most commonly squamous cell carcinomas (SCC), that typically arise from the epithelial lining of the oral cavity, sinonasal tract, pharynx, larynx and salivary glands.^1^ Most HNC are already at an advanced stage when diagnosed, which significantly reduces the survival rate, even after curative treatment.^2^ Major risk factors include tobacco smoking/chewing,^3^ excessive alcohol consumption,^4^ areca (betel) nut, paan masala (Gutkha),^5^ gamma and ultraviolet radiation, overexposure to sunlight, a family history of cancer and increasing age.^6^ The role of human papillomavirus (HPV)^7^ and Epstein–Barr virus (EBV) has also been implicated in the development of oropharyngeal and nasopharyngeal SCC.^3,8^ The global incidence of HNC continues to rise^9,10^ with more than half a million cases annually^11^ and ~12,000 new cases in the UK each year, an increase of 20% in the last decade.^12^ Prognosis remains poor, with a 28–67% chance of survival at five years, depending upon the stage at presentation.^12^

Public health screening/awareness programmes, withdrawal of environmental carcinogens and early detection of precancerous lesions remain the focus for primary and secondary prevention.^3^ However, early detection of some HNC can be difficult due to vague histories and indistinctive diagnostic features. Conventional diagnosis of HNC is based on histopathological evaluation of tissue sections from biopsies or surgical resections, in addition to clinical and radiological examinations. These methods can be time-consuming and are prone to errors in observation or variations in interpretation,^13–15^ which can result in inconsistencies in cancer grading and prognostication.^16^ Consequently, this can cause delays and/or inaccuracies in diagnosis, which can have significant implications on patient management and survival. Indeed, improvements in HNC prediction accuracy and disease outcomes could greatly assist healthcare professionals in the early detection and planning of patient-specific optimal treatments to reduce the disease burden.

### Artificial intelligence: machine learning and medical image analysis

Recent technological advancements in Artificial Intelligence (AI) algorithms, computer hardware and big medical imaging datasets have enabled computer scientists and healthcare researchers to collaborate closely to improve consistency in cancer risk stratification over the use of multi-factor analysis, conventional logistic regression and Cox analyses.^17^

The recent advent of Machine Learning (ML) has seen a surge of interest with the exponential growth of evidence to support its wide applications in a range of cancers.^18–24^ ML is a branch of AI that uses computational methods to detect patterns, gather insight and make predictions about new data by using historical information that has been ‘learnt’. As the volume of training data increases, ML algorithms can produce more accurate and efficient predictions.^25^ Deep learning (DL) is a subfield of ML in which algorithms are structured to create artificial neural networks with multiple hidden layers. These methods have also gained significant popularity in recent years due to their achieving relatively high accuracy of prediction.

**Fig. 1:**
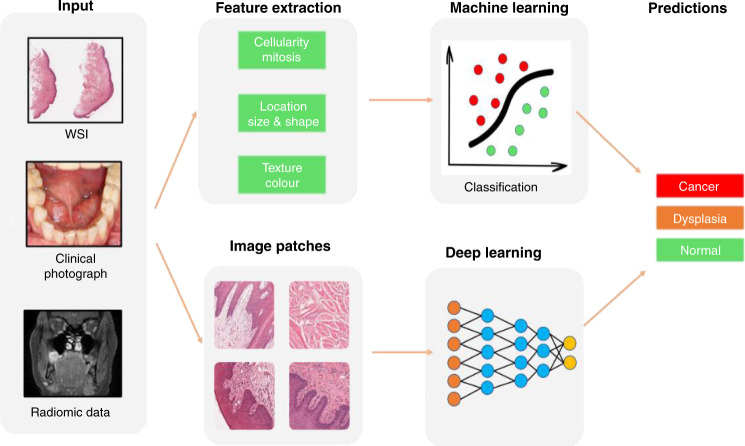
Feature extraction from primary diagnostic imaging modalities to train ML/DL algorithms to aid outcome prediction. Source: The schematic diagrams were prepared in line with the journal’s artwork guidelines; the clinical/histological/radiological images were obtained with appropriate consent from Sheffield Teaching Hospitals NHS Foundation Trust.

Much of the recent focus in cancer diagnostics has centred on digital image analysis and processing, which involves extraction of meaningful information from images to enable delineation of features of clinical interest (segmentation) or description of labels (classification).^26,27^ A number of ad hoc (or hand-crafted) feature analysis-based ML approaches have been shown to be successful in different diagnostic applications, by explicitly defining a prior set of features and processing steps^28^ (Fig. 1). Detection of HNC can be achieved using these ML methods by obtaining clinically important information from primary diagnostic imaging modalities, in which high-dimensional, mineable images can be input to train algorithms. For example, radiomic data can be derived from radiographs, ultrasound (US), computed tomography (CT), magnetic resonance (MR), positron emission tomography (PET) and nuclear medicine imaging methods, such as single-photon emission computed tomography (SPECT). Similarly, histological, cytological and immunohistochemical data can be obtained from high-resolution whole-slide images (WSI) of stained tissue sections from biopsies or surgical resections. Other emerging tools for non-invasive detection of HNC include multispectral narrow-band imaging, Raman spectroscopy, confocal laser endomicroscopy (CLE) and infrared thermal imaging.

This paper seeks to provide an overview of the recently published literature relating to the application of AI/ML methods to aid diagnostic evaluation of HNC. An outline of the different anatomical sites for HNC lesions, the type of diagnostic imaging modality and the AI/ML method used will be presented.

## Methods

### Literature search

Electronic database searches using MEDLINE via OVID, EMBASE and Google Scholar were conducted to retrieve articles published in the English language over the last eleven years (2009–2020). This period was chosen due to the evolving application of AI/ML methods in diagnostic cancer research over the last decade.

The search strategy was developed in collaboration with a medical information specialist (Health Sciences Library, University of Sheffield, UK) to ensure keywords were appropriately chosen for optimal identification of articles. A combination of tailored search strings containing database-specific medical subject headings (MeSH) and controlled vocabulary was used (see Supplementary Information), and grey literature screened. Whilst not intended as a formal systematic review, the recommendations of the Preferred Reporting Items for Systematic Reviews and Meta-Analyses (PRISMA) statement and checklist were followed where possible.

### Study selection

The selection criterion was jointly developed by the authorship team. The principal inclusion criteria were studies applying AI/ML methods to aid diagnosis of HNC using image analysis, with no restrictions placed on the types of methods or imaging modalities used. Due to the anticipated small number of studies in the field, a broad range of studies such as those using AI/ML to identify or differentiate between benign/pre-malignant/malignant pathology, classify disease subtype, segment cancer regions or predict disease outcome.

Studies using AI/ML to predict cancer susceptibility, metastasis, recurrence, survival or treatment efficacy were not included in this review. Studies focussing solely on the evaluation of oesophageal or thyroid cancers were excluded, unless they were included as part of a larger study that included other HNC lesions. Narrative reviews, letters to editors, commentaries, conference abstracts and animal studies were also excluded. All articles were independently screened by two authors (H.M. and M.S.). The first screen involved the assessment of study title and abstracts and the removal of duplicate articles. The second screen involved comprehensive full-text examination against the predefined criterion. In the case of author discrepancy, two further authors (NMR, SAK) were consulted to make a final decision on article inclusion.

### Data capture and synthesis

Relevant data were extracted, tabulated and processed in Microsoft Excel^®^ (Microsoft Corporation, Washington, USA). Data collection included

- Study details (date of publication, authors, study location and aims)
- Study methods (anatomical sites for HNC lesions, diagnostic imaging modality, dataset sizes and application of AI/ML methods to aid cancer diagnosis/outcome)
- AI/ML algorithm performance (reported accuracy measures)

A narrative synthesis with the relevant graphical display is presented. Due to the variations in study outcomes and heterogeneity of data, a meta-analysis for the calculation of adjusted pool estimates was not performed.

## Results

The electronic database search retrieved 771 scientific articles. After the removal of duplicates and screening of study titles and abstracts, 698 articles were excluded. Detailed full-text examination of remaining articles excluded a further 41 studies, resulting in 32 articles for inclusion (see Supplementary Table). Among the selected articles, 9 were published between 2009 and 2014, and the remaining 23 articles published between 2015 and 2020. The primary outcomes of interest were anatomical sites of the HNC lesions, diagnostic imaging modalities used for algorithm training/optimisation and the type of AI/ML method.

### Anatomical sites for HNC lesions

**Fig. 2:**
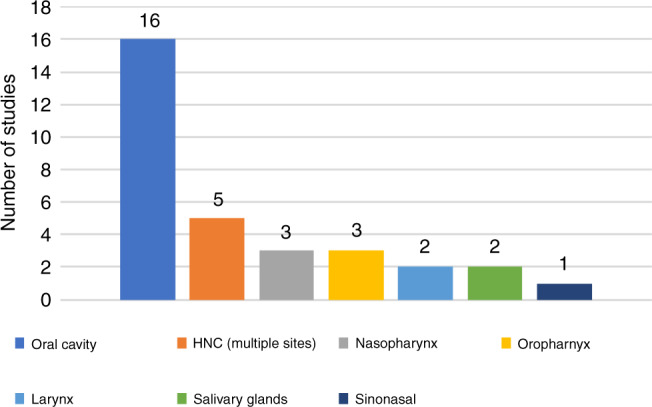
HNC anatomical site distribution. A bar chart showing the proportion of included studies based on head and neck cancer anatomical subtype.

Figure 2 illustrates the different anatomical sites for the HNC lesions in the selected studies, with the largest proportion (16 studies) involving the oral cavity. Amongst these, nine studies focussed on the assessment of oral squamous cell carcinoma (OSCC) and seven studies focussed on the evaluation of, or differentiation between, oral potentially malignant disorders (OPMD) and OSCC. The remaining studies focussed on assessment of nasopharyngeal SCC (*n* = 3),^29–31^ laryngeal SCC (*n* = 2),^32,33^ oropharyngeal SCC (*n* = 3),^34–36^ parotid gland neoplasms (*n* = 2)^37,38^ and differentiation between sinonasal SCC from inverted papilloma (*n* = 1).^39^ In four studies,^40–42^ tissue sections of HNC from various different sites (tongue, floor of mouth, soft palate, mandible, gingivae, alveolar ridge, supraglottis, maxillary sinus, nose, thyroid and parotid gland) were evaluated. One of these studies did not specify the anatomical sites of the HNC lesions.^43^

### Diagnostic imaging modalities for algorithm training/optimisation

**Fig. 3:**
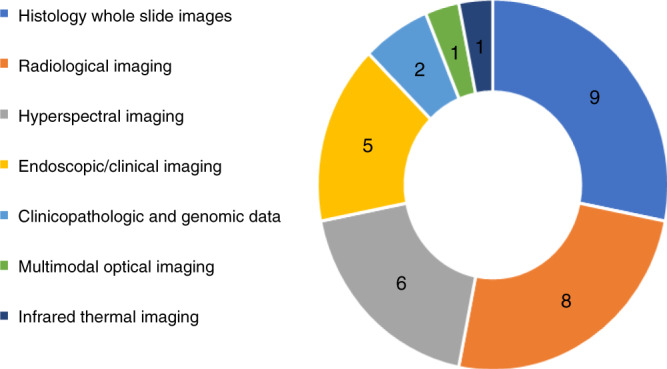
Type of imaging modality/input data used. A pie chart showing the proportion of identified studies based on the type of maging modality/input data used to train AI/ML algorithms.

Histology WSI of haematoxylin and eosin (H&E)-stained tissue sections was used to train AI/ML algorithms in nine studies (Fig. 3). Radiology image data were used in eight studies and obtained from dynamic contrast-enhanced MRI (DCE-MRI) (*n* = 3),^31,39,43^ CT (*n* = 2),^36,37^ PET/CT (*n* = 1),^29^ US (*n* = 1)^38^ and plain film intraoral radiographs (*n* = 1).^44^ Other imaging modalities included hyperspectral imaging (HSI) (*n* = 6), endoscopic/clinical imaging (*n* = 5), infrared thermal imaging (*n* = 1)^45^ and multimodal optical imaging (*n* = 1).^46^ In the remaining two studies, clinicopathologic, genomic and exfoliative cytological data were used to predict outcomes based on traditional statistical analysis methods.^47,48^

### Histology whole-slide imaging

In nine studies, histology WSI was used to develop algorithms for evaluation of OSCC (*n* = 2),^49,50^ OPMD (*n* = 4),^51–54^ laryngeal SCC (*n* = 1),^32^ oropharyngeal SCC (*n* = 1)^35^ and multiple HNC sites (*n* = 1).^55^

These studies used a variety of different ML approaches to delineate specific histological features of interest with downstream statistical analysis to compare differences in spatial architectural patterns for differentiation between benign and malignant lesions. ML tools were developed to assess differences in detection of quantity, geometry, compactness and eccentricity of sub-epithelial connective tissue cells and to identify textural differences between normal and oral submucous fibrosis tissue (with and without dysplasia or atrophy) using approaches including Brownian motion.^51–53^

In one study, unsupervised ML methods were used for the automated identification of tissue compartments in oropharyngeal SCC (OPSCC) tissue microarrays.^35^ Morphological measurements of cell and nuclei were used for the classification of epithelial and stromal tissue achieving a pixel-level F1 score of 80–81%. A further study showed that stimulated Raman scattering histology integrated with DL algorithms provided the good potential for delivering a rapid intraoperative diagnosis of laryngeal SCC with an accuracy of 90%.^32^ Findings showed that this method could identify tissue neoplasia at the simulated resection margins that appear grossly normal with the naked eye, highlighting the potential to enhance surgical resection and reduce disease recurrence.

A recent systematic review highlights emerging evidence to support the role of ML methods for histology images as a potentially useful diagnostic aid for the detection of OSCC and some OPMD, but identifies a lack of evidence for other head and neck precancerous or cancerous lesions.^44^ However, the overall quality of evidence in these studies is low, mainly due to the use of small unicentric datasets and a high risk of bias that could have overestimated model accuracy rates.

### Radiological imaging

Three studies used radiomic-based feature prediction from MRI to aid assessment of various HNC lesions,^43^ including nasopharyngeal^31^ and sinonasal SCC.^39^ Deng et al.^43^ proposed an automatic segmentation method using traditional ML techniques for evaluation of HNC lesions demonstrating superior segmentation performance (area overlap measure of 0.76 + /−0.08 and accuracy of 86 ± 8%), which outperformed previous similar studies. Huang et al.^31^ evaluated the performance of two region-based segmentation methods for the evaluation of nasopharyngeal SCC. Ramkumar et al.^39^ found that MRI-based textural analysis had the potential to differentiate sinonasal SCC from inverted papilloma (accuracy 89.1%) with results comparable to manual assessment by neuroradiologists (*P* = 0.0004).

Three studies used CT-based textural analysis for the evaluation of HNC. Ajmi et al.^37^ developed an approach using spectral dual-energy CT (DECT) data from multi-energy virtual monochromatic image datasets to capture the energy-dependent changes in tissue attenuation for the classification of common benign parotid gland neoplasms (Warthin tumour and pleomorphic adenoma) with an accuracy of 92%. Whereas Ranjbar et al.^36^ used CT-based texture analysis to classify the HPV status of oropharyngeal SCC (accuracy 75.7%). In another study, Wu et al.^29^ developed an automated algorithm for the identification of nasopharyngeal SCC on PET/CT examination with 100% accuracy for detection of hypermetabolic lesions larger than 1 cm in size.

Only one study used textural features derived from an ultrasound (US) using radio-frequency echo signals and image data to enable automatic differentiation between malignant and benign parotid gland lesions (accuracy 91%) based on a supervised classification system.^38^ In another study, gravitational search-optimised echo-state neural networks were developed for early prediction of OSCC from intraoral X-rays with a detection accuracy of 99.2%.^56^

### Endoscopic/clinical imaging

Four studies used clinical data from endoscopic imaging for the detection of oral,^57^ nasopharyngeal,^30^ oropharyngeal^34^ and laryngeal cancers.^33^ Amongst these, two studies employed DL methods. The first study developed algorithms for early detection of nasopharyngeal malignancies (accuracy of 88.7%)^30^ providing surgeons with useful biopsy guidance. The second study demonstrated the superior performance of DL compared to textural feature-based ML methods (accuracy 88.3%, sensitivity 86.6% and specificity 90%) in recognition of sub-surface microanatomical in vivo cell structures using confocal laser endomicroscopy (CLE) in OSCC patients.^56^

In two studies, traditional ML approaches were used for the detection of oropharyngeal and laryngeal SCC. Mascharak et al.^34^ used multispectral narrow-band imaging and white-light endoscopy (WLE) to quantify the lymphoepithelial tissues of the oropharynx. The results showed a promising ability to differentiate between oropharyngeal SCC and healthy mucosa based on differences in colour (accuracy 65.9% compared to 52.3% under WLE, *P* = 0.0108), presumably a reflection of surface angiogenic and local inflammatory changes. Whereas Moccia et al.^33^ used traditional ML techniques to classify laryngeal tissues (normal vs malignant) in narrow-band endoscopic images by exploiting textural information (classification recall 93–98%). These studies demonstrate a promising step towards an endoscope-based processing system to support the early-stage diagnosis of HNC lesions that may go unnoticed by the human eye.

Song et al.^58^ developed a smartphone-based intraoral dual-modality imaging platform to classify OPMD and malignant lesions based on autofluorescence and white-light images using a convolutional neural network (CNN). The results demonstrated an accuracy of ~86.9%, although the training sample was relatively small (66 normal samples and 64 suspicious). Other limitations, including training the CNN algorithms on tissue from different anatomical regions (for normal, dysplastic and malignant tissues), are likely to exhibit different autofluorescence characteristics due to the varying tissue structural and biochemical compositions.

### Other imaging modalities

Six studies used HSI for AI/ML training. Three of those studies focussed on early detection and diagnosis of OSCC. Liu et al.^59^ used HSI to measure the reflectance spectra in the tongue to enable differentiation between normal and cancerous tissue, with a recognition rate of 96.5%. Roblyer et al.^46^ used multispectral wide-field optical imaging—which included white-light reflectance, autofluorescence, narrow-band reflectance and cross-polarised imaging modalities—to distinguish between oral cancer/precancer and non-neoplastic mucosa by evaluating image contrast. Their results showed that autofluorescence imaging at 405-nm excitation wavelength provided the greatest image contrast, and the ratio of red-to-green fluorescence intensity computed from these images provided the best classification of dysplasia/cancer versus non-neoplastic tissue (sensitivity of 100%, specificity of 85%). Although this approach accurately distinguished malignant from benign tissue, the ability to separate precancerous lesions from cancers was found to be limited. In the third study, Quang et al.^60^ also used multimodal optical imaging, in which autofluorescence imaging was used to identify high-risk regions within the oral cavity, followed by high-resolution microendoscopy to confirm or exclude the presence of neoplasia (defined by the authors as diagnoses of moderate dysplasia or worse). Data from 92 sites (*n* = 30) were used to develop algorithms for the automatic identification of OSCC in vivo. Diagnostic performance was evaluated prospectively using images from 114 sites (*n* = 70) and the confirmed histological diagnosis based on either a biopsy or an excised surgical specimen. Amongst the sites that were biopsied (*n* = 56), the classification accuracy for detection of benign and cancerous lesions was 100 and 85%, respectively. Amongst the sites that corresponded to a surgical specimen (*n* = 58), multimodal imaging correctly classified 100% of benign and 61% of neoplastic sites.

Jeyaraj et al.^45^ developed a convolutional neural network (CNN) classifier for OSCC detection using multidimensional HSI (accuracy of 91.4%). Similarly, Halicek et al.^41^ also developed a CNN classifier that was trained using HSI of HNSCC surgical specimens, including thyroid cancer, and normal head and neck tissue samples. This model showed the potential to produce near-real-time automatic tissue labelling for intraoperative cancer detection using HSI.

One study explored the viability of digital infrared thermal imaging (DITI) for screening and detection of OSCC.^61^ DITI is a non-invasive, non-ionising, radiation hazard-free modality that essentially produces a heat map of an object by capturing its radiated thermal energy. The authors developed a semi-automated screening framework for OSCC by extracting significantly discriminating textural features from facial thermograms for classification into normal, precancerous or malignant categories achieving an accuracy rate of 85.42%.

Another study developed diagnostic algorithms for HNSCC detection using ML constructed by mass spectra obtained from non-cancerous (*n* = 15, 114 mass spectra) and HNSCC (*n* = 19, 141 mass spectra) specimens by probe electrospray ionisation mass spectrometry. The clinical validity of this approach was evaluated to discriminate tumour-specific spectral patterns using intraoperative specimens of HNSCC and normal mucosa with positive and negative-ion modes showing accuracies in HNSCC diagnosis of 90.48% and 95.35%, respectively.

In another study,^48^ exfoliative cytology, histopathology and clinical data of normal subjects (*n* = 102), oral leukoplakia (OLK) patients (*n* = 82) and OSCC patients (*n* = 93) were collected for quantitative risk stratification of OLK lesions. This involved expert-guided data transformation and reconstruction for automatic data processing to reveal a risk index for OSCC prediction (sensitivity: median >0.98, specificity: median >0.99).

### Type of AI/ML method

**Fig. 4:**
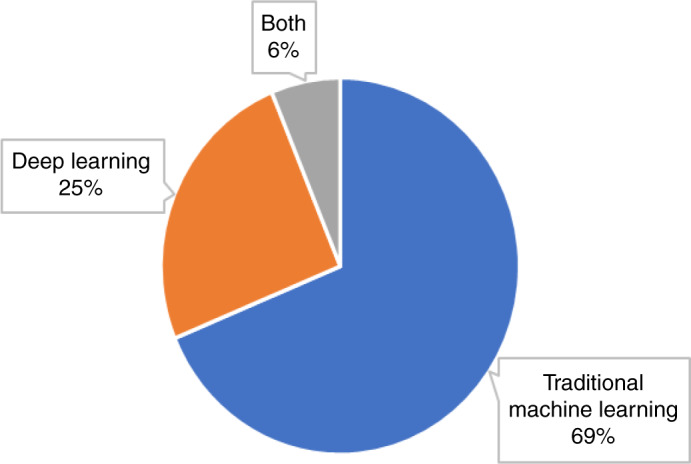
Types of AI methods used. Proportion of studies using traditional ML methods, modern deep learning methods and a combination of both types of methods.

Figure 4 illustrates the proportion of AI methods used in the selected studies. Traditional ML methods were employed in 22 studies (69%) with common approaches, including Support Vector Machine, Logistic Regression, Random Forest, Decision Tree, *K*-Nearest Neighbour, Bayesian Classifier and Linear Discriminant Analysis. DL-based neural networks were employed in eight studies (25%), and a combination of traditional ML and DL methods were used in two studies (6%).

**Fig. 5:**
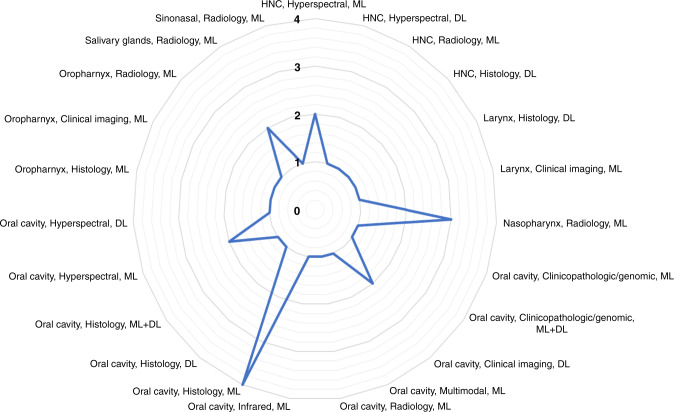
Overview of recently published literature. A diagram illustrating an overview of the recently published literature based on anatomical site of lesion, imaging modality and AI methods used.

Figure 5 provides a breakdown of the selected studies by combining the anatomical site of lesion, imaging modality and AI methods used in the recently published literature. Traditional ML methods were most frequently used for the detection of precancerous or cancerous lesions of the oral cavity (11 studies), and specifically in studies using histology WSI (four studies). DL methods were used for detection of HNSCC lesions of the oral cavity (five studies), nasopharynx (one study), larynx (one study) and various other head and neck sites as specified in a study by Halicek et al.^41^

## Discussion

This paper provides an insight into the recent application of AI/ML for the evaluation of HNC lesions using digital image analysis. It has shown, primarily, a wide breadth of imaging modalities that have been used to retrieve input data for algorithm training in the last decade. Whilst a detailed statistical analysis of the heterogeneous dataset samples has not been undertaken, most studies have indicated that ML can achieve high degrees of accuracy and precision that can exceed the abilities of standard statistical techniques and human judgement in making predictions about data. This supports seminal claims made by Meehl in 1954,^62^ and more recent meta-analyses,^63,64^ which propose that correctly used mechanical (i.e., algorithmic) methods make more efficient and reliable decisions and predictions about patient outcomes and treatment. However, despite findings highlighted by our paper, very few ML tools have actually been deployed into clinical practice.

Whilst a formal risk of bias analysis has not been conducted for the cited studies, the reported accuracy rates should be interpreted with caution. This is because most studies have used small unicentric datasets that may be biased towards a particular patient demographics. Multi-centric research will inevitably allow a more diverse dataset with the inclusion of patients from different geographical locations, populations and demographics that will enhance algorithm performance by incorporating biological and technical variance.

Findings demonstrate the greatest proportion of studies to have evaluated the detection of OPMD and cancerous lesions within the oral cavity (Fig. 2) with histology WSI and radiological imaging being the most frequently used modalities for algorithm training (Fig. 3). This is consistent with the increasing ubiquity of digital slide scanners in pathology laboratories and the emergence of radiomics that has broadened the scope of routine medical imaging in clinical oncology.

With the continued evolution of AI algorithms and computational power, a plethora of computational methods has been developed for fast and reproducible diagnosis and prognosis of HNC, as exemplified in this paper. The emergence of various high-resolution imaging modalities (i.e., multimodal optical, microendoscopic, hyperspectral and infrared thermal) has provided an unprecedented opportunity for quantitative feature extraction by conversion into mineable images at relatively low cost and non-invasively. Having said this, histology WSI remains the most superior imaging modality for data leverage. This is because each image provides multi-gigapixel-level information, thereby resulting in hundreds of thousands of sub-images (image patches) per WSI for analysis and algorithm training.

Early work has been largely based on the development and application of traditional ML methods (Fig. 4); however, in recent years, the use of DL for HNSCC diagnosis and prognostication has evolved. This opens the opportunity to develop state-of-the-art DL techniques that can be combined with traditional approaches to improve detection accuracy of head and neck precancerous and cancerous lesions, as well as predict the course of a precancerous or cancerous lesion learning from retrospective data. Another exciting research avenue would be the development of new data fusion algorithms that combine imaging modalities such as radiologic, histologic and molecular measurements to aid disease detection, classification and outcome prediction.

## Supplementary information

Supplementary information

Supplementary Table 1

## Data Availability

All data generated or analysed during this study are included in this published article and its supplementary information files.
