# Supplementary material for: Artificial Intelligence-based methods in head and neck cancer diagnosis: an overview
Source: Br J Cancer. 2021 Apr 19;124(12):1934–40. doi: 10.1038/s41416-021-01386-x (PMC8184820; doi:10.1038/s41416-021-01386-x)
Supplement: Supplementary file 1 — Supplementary information [file 41416_2021_1386_MOESM1_ESM.docx]

**Ovid MEDLINE search strategy**

1. artificial intelligence.mp. or exp Artificial Intelligence/

2. machine learning.mp. or Machine Learning/

3. deep learning.mp. or Deep Learning/

4. Image Processing, Computer-Assisted/ or automated detection.mp. or Diagnosis, Computer-Assisted/

5. "Neural Networks (Computer)"/ or neural networks.mp.

6. automated image analysis.mp.

7. digital image analysis.mp.

8. 1 or 2 or 3 or 4 or 5 or 6 or 7

9. Mouth Neoplasms/ or oral epithelial dysplasia.mp. or Leukoplakia, Oral/

10. oral leukoplakia.mp.

11. oral neoplasm.mp.

12. oral precancer.mp.

13. oral cancer.mp.

14. "head and neck cancer".mp. or "Head and Neck Neoplasms"/

15. "head and neck malignancy".mp.

16. 9 or 10 or 11 or 12 or 13 or 14 or 15

17. Diagnosis/ or diagnosis.mp.

18. diagnostic performance.mp.

19. 17 or 18

20. 8 and 16 and 19

21. limit 20 to (english language and humans and last 10 years)
