# Supplementary material for: Artificial Intelligence-based methods in head and neck cancer diagnosis: an overview
Source: Br J Cancer. 2021 Apr 19;124(12):1934–40. doi: 10.1038/s41416-021-01386-x (PMC8184820; doi:10.1038/s41416-021-01386-x)
Supplement: Supplementary file 2 — Supplementary Table 1 [file 41416_2021_1386_MOESM2_ESM.docx]

| AI Method | Study Reference | AI/ML Method | Imaging Modality & Dataset | Reported Diagnostic Performance |
| --- | --- | --- | --- | --- |
| ML | **Ranjbar *et al.* (2018)** | Quadratic discriminant analysis | Contrast-enhanced neck CT images (HPV positive oropharyngeal SCC n=92, HPV negative oropharyngeal SCC n=15) | Classification of HPV Status of Oropharyngeal SCC:   - Accuracy 75.7% |
|  | **Ajmi *et al.* (2018)** | Random Forest | Dual-energy CT images (n=42) | Classification of benign parotid tumours:   - Accuracy 92% - Sensitivity 86% - Specificity 100% |
|  | **Wu *et al.* (2012)** | Support Vector Machine | PET/CT images (n=25) | Classification of nasopharyngeal SCC:   - Sensitivity 99.3% - False Positive 4.8% |
|  | **Mascharak *et al.* (2018)** | Naive Bayes Classification | Multispectral images (n=20) | Detection of Oropharyngeal SCC:   - Accuracy 65.9% - Sensitivity 66.8% - Specificity 64.9% |
|  | **Moccia *et al.* (2017)** | Support Vector Machine | Endoscopic videos (n=33) | Laryngeal tissue classification:   - IQR 93-98% |
|  | **Lu *et al.* (2017)** | Quadratic Discriminant Analysis | Histology tissue microarrays (n=115) | Classification of nuclear morphology:   - Accuracy 87% - AUC 72% |
|  | **Baik *et al.* (2014)** | Random Forest | Histology WSI (normal n=29, carcinomas in situ n=5, OSCC n=28). | Classification of oral premalignant lesions:   - Accuracy 80% - Sensitivity 80.6% - Specificity 79.3% |
|  | **Krishnan *et al*. (2012)** | Decision Tree, K-NN, GMM | Histology WSI (oral sub-mucous fibrosis n=42, normal n=10) | OSCC identification:   - Accuracy 95.7% - Sensitivity 94.5% - Specificity 98.8% |
|  | **Krishnan *et al.* (2009)** | Support Vector Machine | Histology WSI (n=1840 cells from OSF and normal oral tissue) | Classification of cells:   - Accuracy 88.69 |
|  | **Quang *et al.* (2017)** | Linear Discriminate Analysis | Multimodal optical images (n=206) | OSCC Detection:   - Nonneoplastic Accuracy 100% - Neoplastic Accuracy 85% |
|  | **Fei *et al.* (2017)** | Linear Discriminate Analysis | Hyperspectral images (n=15) | Tumour margin segmentation:   - Accuracy 90% - Sensitivity 89% - Sensitivity 95% |
|  | **Ashizawa *et al.* (2017)** | Logistic Regression | Mass spectra with positive- and negative-ion modes of probe electrospray ionization mass spectrometry images (n=255) | HNSCC diagnosis:   - Positive-ion Accuracy 90.48% - Negative-ion Accuracy 95.35% |
|  | **Fouad *et al.* (2017)** | Clustering | Tissue micro-arrays (n=50) | Segmentation of oropharyngeal SCC:   - F1-score: 0.81 |
|  | **Liu *et al.* (2012)** | Dictionary Learning | Hyperspectral images (n=65) | OSCC (tongue) detection:   - Accuracy 96.5% |
|  | **Lu *et al.* (2017)** | LDA, QDA, SVM, SVM-RBF | Hyperspectral images (n=36) | Detection of HNSCC:   - Sensitivity 91% - Specificity 91% |
|  | **Roblyer *et al.* (2010)** | Linear Classifier, Decision Tree | Narrowband reflectance, autofluorescence, and polarised  Reflectance images (n=72) | Classification into non-neoplastic, dysplastic, or cancer:   - Sensitivity 100% - Specificity 85% |
|  | **Chakraborty *et al.* (2016)** | K-Means Guided Cluster Prototype Classifier | Infrared images (malignant n=81,  precancerous n=59, normal n=63). | Classification into malignant, precancerous or normal class:   - Accuracy 85.31% |
|  | **Deng *et al.* (2017)** | Support Vector Machine | Dynamic Contrast-Enhanced  MRI images (n=120) | Segmentation of HNSCC:   - Corresponding ratio 79% - Percent match 86% |
|  | **Ramkumar *et al.* (2017)** | Diagonal Linear Discriminate Analysis, Support Vector Machines, and Diagonal Quadratic Discriminate Analysis | MRI images of inverted papilloma and SCC (n=46) | Classification of SCC and inverted papilloma:   - Accuracy 84.6% - Sensitivity 85.7% - Specificity 83.3% |
|  | **Huang *et al.* (2013)** | Similarity learning via supervised spectral clustering | MRI of nasopharyngeal SCC images (n=253) | Nasopharyngeal SCC Segmentation:   - Sensitivity 98% - Positive predictive value 78% |
|  | **Siebers *et al.* (2010)** | Texture feature based maximum likelihood classifier | Ultrasound radio-frequency echo data of parotid gland lesions (n=138) | Parotid Gland Lesions:   - AUC 91% |
|  | **Al-Ma'aitah *et al.* (2018)** | Gravitational search  optimized echo state neural networks | X-ray images (dataset size not reported) | OSCC Prediction:   - Accuracy 99.2% - Specificity 96.5% - Sensitivity 96% |
| DL | **Bofan *et al.* (2018)** | Convolutional Neural Network (VVG-16) | Autofluorescence and white light images (n=190) | Oral dysplasia classification:   - Accuracy 86.9% - Sensitivity 85% - Specificity 88.7% |
|  | **Aubreville *et al.* (2017)** | Convolutional Neural Network (Inception-v3) | Confocal Laser Endomicroscopic image sequences (n=7894) | OSCC Classification:   - Accuracy 88.3% - Sensitivity 86.6% - Specificity 90.0% - AUC 95.5% |
|  | **Li *et al.* (2018)** | Convolutional Neural Network (Inception) | Endoscopic images (n=28,966 training set, n=1430 testing set) | Detection of Nasopharyngeal SCC:   - Accuracy 88.7% |
|  | **Mookiah *et al.* (2011)** | Neural Network | Histology WSI (n=112) | Classification of HNSCC:   - Accuracy 96.43% - Sensitivity 92.31% - Specificity 100% - AUC 96.15% |
|  | **Zhang *et al.* (2019)** | Convolutional Neural Network (ResNet-34) | Fresh surgical specimens post resection (n=80) | Classification of laryngeal SCC:   - Accuracy 90% - AUC 95% |
|  | **Halicek *et al.* (2017)** | Convolutional Neural Network (AlexNet) | Hyperspectral images of cancer specimen margins (n=88) | Classification of HNSCC:   - Accuracy 96.4% - Sensitivity 96.8% - Specificity 96.1% |
|  | **Jeyaraj *et al.* (2019)** | Convolutional Neural Network (Inception-v3) | Hyperspectral images (n=500) | Classification of malignant and benign cancer:   - Accuracy 91.4 - Sensitivity 94% - Specificity 91%   Classification of malignant cancer and normal Tissue:   - Accuracy 94.5% |
|  | **Halicek *et al.* (2019)** | Convolutional Neural Network (Inception-v4) | Histology WSI (n=381) | SCC Detection:   - AUC 91.6%   Thyroid Carcinoma   - AUC 95.4% |
| ML & DL | **Liu *et al*. (2015)** | SVM, Neural Network, Random Forest | Exfoliative cytology, histopathology and clinical data (oral leukoplakia n=82, OSCC N=93, control n=102) | Oral leukoplakia Classification:   - Sensitivity 98% - Specificity 99% |
|  | **Das *et al.* (2018)** | CNN + Random Forest | Histology WSI (n=126) | Epithelial Layer Segmentation:   - Accuracy 98.42% - Sensitivity 97.76%   Keratin Pearl Detection:   - Accuracy 96.88% |

***Supplementary table***
